# Supplementary material for: Impact of Gut Dysbiosis on the Risk of Non-Small-Cell Lung Cancer
Source: Int J Environ Res Public Health. 2022 Nov 30;19(23):15991. doi: 10.3390/ijerph192315991 (PMC9740010; doi:10.3390/ijerph192315991)
Supplement: Supplementary file 1 [file ijerph-19-15991-s001.zip › ijerph-2039643-supplementary.pdf]

Figure S1. Alpha diversity (Chao 1, ACE and Fisher) analysis in the NSCLC cases and normal controls. Alpha diversity analysis using Chao1 (A), ACE (B), and FISHER (C) indexes of each sample in the NSCLC cases and normal controls. The data are presented as the mean ± SD. p<0.05 was considered to be statistically significant.

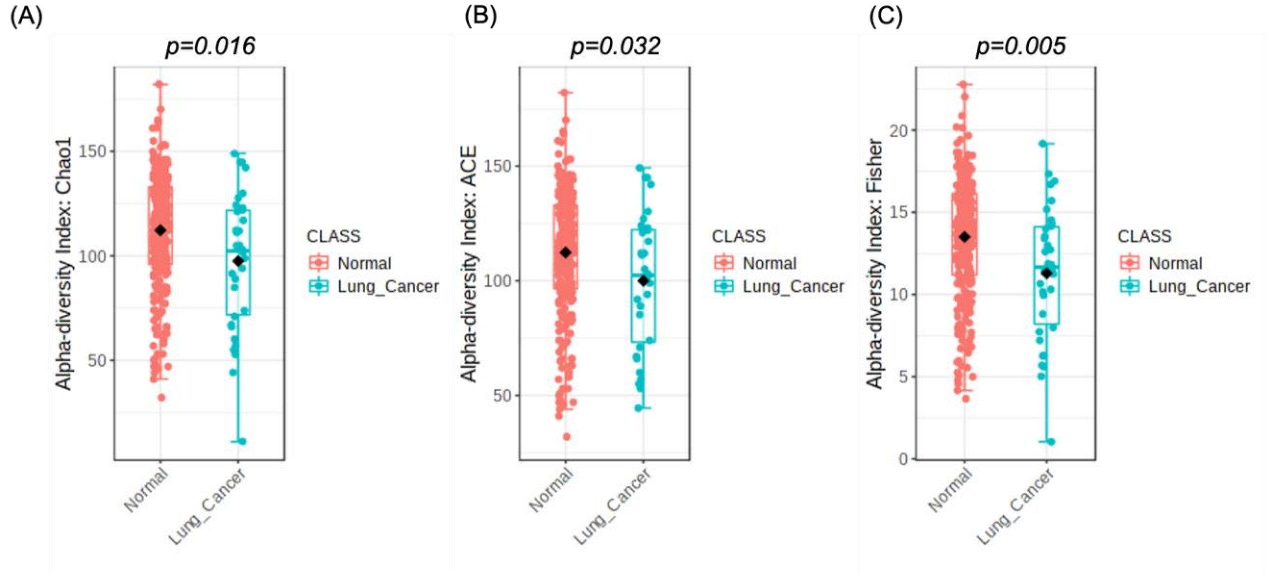

Figure S2. Top 10 OTU abundances at phylum and genus levels of the gut microbes in the NSCLC cases and normal controls. Bar plot at phylum (A) and genus (B) levels of the top 10 most abundant gut microbes showed changed in the NSCLC patients compared with the normal controls.

A.

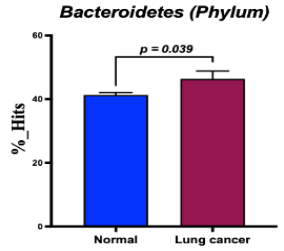

B.

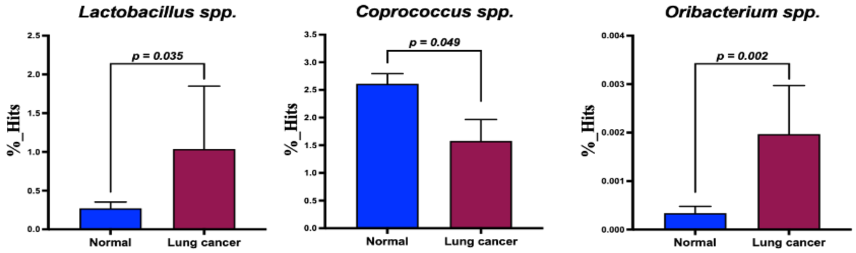

Figure S3. Species levels of the gut microbial biomarkers determined by heatmap analysis were distinct between NSCLC cases and normal controls. Bar plot at the species level of the core gut microbes determined by the heatmap were altered in the NSCLC patients compared with the normal controls.

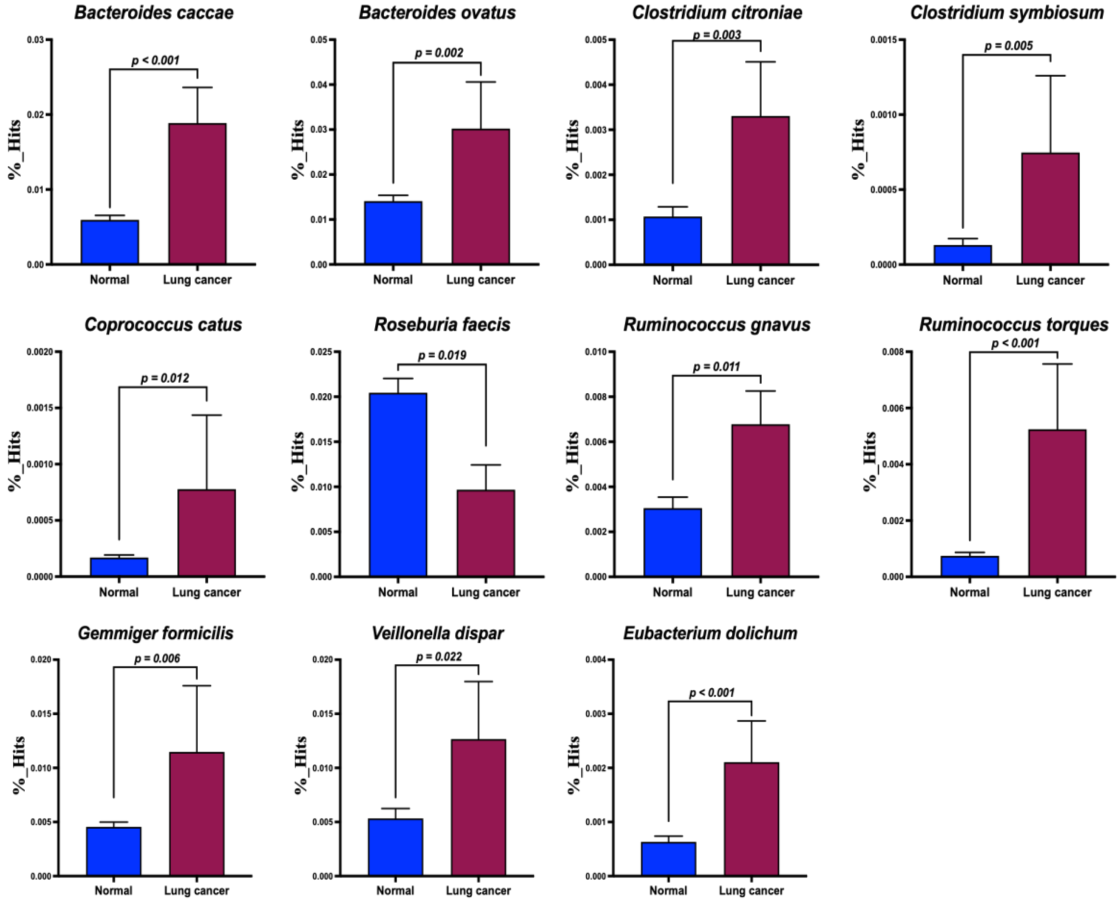

Figure S4. NSCLC-associated gut dysbiosis was not affected by metabolic disorders (diabetes, cardiovascular disorders, and hypertension) or chronic obstructive pulmonary disease (COPD). There were no significant changes in the core gut microbes with regards to metabolic disorders (A) and COPD (B).

A. Metabolic diseases: DM (n=4), Hypertension (n=8)

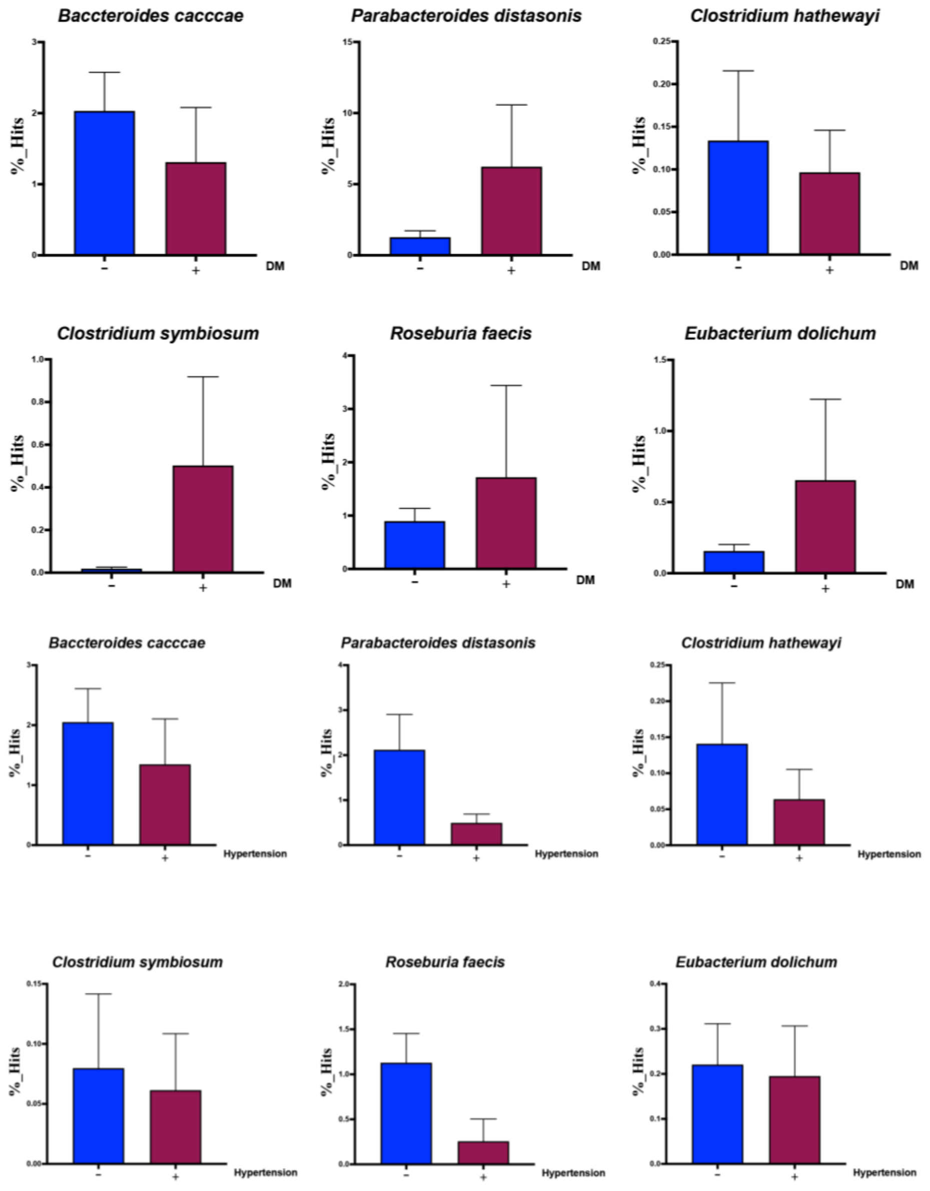

B. COPD (n=5)

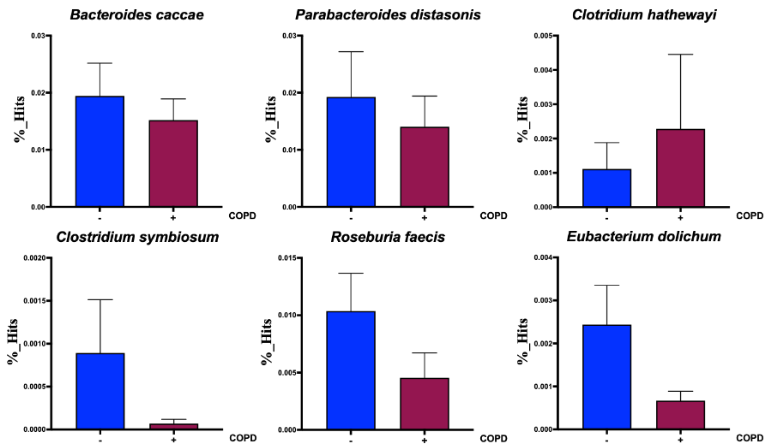

Figure S5. Critical gut microbes involved in NSCLC risk were analyzed by random forest analysis. To detect unique OTU markers of NSCLC, we conducted five-fold cross-validation using a random forest model between 34 patients with NSCLC and 268 normal controls in the discovery set. The top 10 OTUs markers were selected as the optimal marker set by random forest model

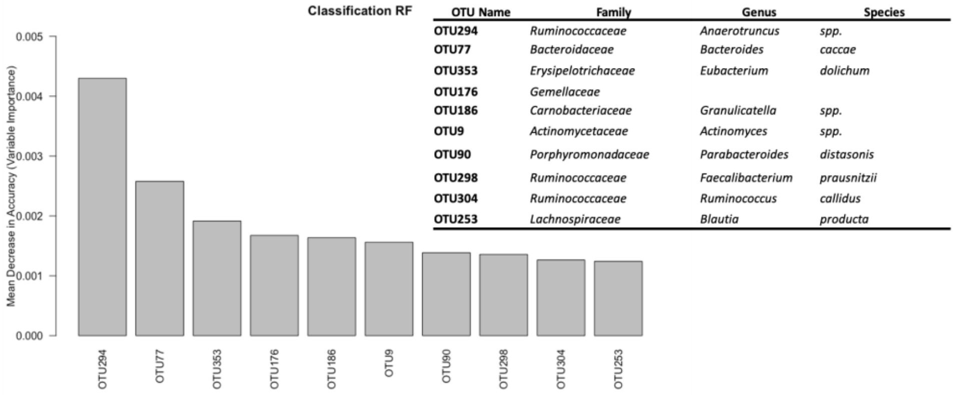

OTU Name	Family	Genus	Species
OTU294	*Ruminococcaceae*	*Anaerotruncus*	*spp.*
OTU77	*Bacteroidaceae*	*Bacteroides*	*caccae*
OTU353	*Erysipelotrichaceae*	*Eubacterium*	*dolichum*
OTU176	*Gemellaceae*		
OTU186	*Carnobacteriaceae*	*Granulicatella*	*spp.*
OTU9	*Actinomycetaceae*	*Actinomyces*	*spp.*
OTU90	*Porphyromonadaceae*	*Parabacteroides*	*distasonis*
OTU298	*Ruminococcaceae*	*Faecalibacterium*	*prausnitzii*
OTU304	*Ruminococcaceae*	*Ruminococcus*	*callidus*
OTU253	*Lachnospiraceae*	*Blautia*	*producta*
